# Challenging the status quo: A guide to open and reproducible neuroimaging for early career researchers

**DOI:** 10.1162/IMAG.a.21

**Published:** 2025-06-24

**Authors:** Nikhil Bhagwat, Sebastian Urchs, Jean-Baptiste Poline, Yu-Fang Yang

**Affiliations:** Montreal Neurological Institute & Hospital, McGill University, Montreal, QC, Canada; Division of Experimental Psychology and Neuropsychology, Department of Education and Psychology, Freie Universität Berlin, Berlin, Germany

**Keywords:** open science, reproducible research, neuroimaging, MRI, EEG, early career researchers, neuroinformatics

## Abstract

In the last decade, neuroimaging research has seen a proliferation of open tools, platforms, and standards aimed at addressing the reproducibility crisis in the field. The growing awareness on this topic is bringing about a cultural shift in the scientific community, especially among early career researchers (ECRs). As members of this demographic, we can attest to the fact that the adoption of these new tools and practices remains a challenge. This work aims to provide a practical guide for ECRs to navigate the expanding landscape of the open-science resources and make proactive decisions for their research workflows dealing with large, multiple datasets. From our own experience, we describe the common hurdles faced in typical research workflow and provide a set of solutions that could serve as a starting point for researchers looking for practical tools and protocols. Through a hypothetical scenario, we walk through the steps of curating, processing, harmonizing, and publishing a dataset while describing the tools and practices helpful for adopting FAIR (findable, accessible, interoperable, and reusable) principles. We hope this guide can help ECRs and others to simplify their daily research life as we all strive towards more open, reproducible, and translational neuroscience research.

## 1 Introduction

Wait, do not roll your eyes—this is not yet another*Call for High-level Advocacy of Open Science (CHAOS),*but more of an exercise in self-reflection to create a set of lessons learned and guidelines that help newly minted early career researchers (ECRs) in the field of neuroimaging. For 15–20 years, numerous meta-studies, opinion-, and 10-simple-rules-articles have underscored the need for open and reproducible science practices, and the adoption of findable, accessible, interoperable, and reusable (FAIR) data principles for instilling trust and ensuring the reliability of our research endeavours (Forscher, 1963;Guide for Reproducible Research — the Turing Way, n.d.;Marić et al., 2022;Niso et al., 2022;Prlić & Procter, 2012;Sandve et al., 2013;Wagner et al., 2022;Wilkinson et al., 2016). Yet, the adoption of these much-needed scientific practices is challenged by technical and cultural complexities as well as the lack of academic incentives and communication confounded with*CHAOS*. Having recently muddled and struggled through these hurdles, we believe it would be therapeutic to ourselves, and helpful to other ECRs alike, to go through an exercise of introspection. Broadly, our retrospective lessons can help the laboratories and institutes rehabilitate research data management plans and to bypass future scientific grief. Personally, we hope this effort will serve as a helpful guide to ECRs navigating the*CHAOS*full open-science landscape while balancing un-*FAIR*incentives for professional success.

We begin by admitting the problem—*open*and*reproducible*science is a complex endeavour that is hard to define, facilitate, and incentivize. While there are many well-developed technical solutions, their adoption in a laboratory depends on their user-friendliness, availability of training resources, and whether the time investment in these tools is valued within one’s research ecosystem. Even for researchers with technical expertise, keeping up with an ever-growing set of tools can be overwhelming, and often the priorities skew towards the quantity of scientific output rather than the quality of methodology. Consequently, in highly competitive academic research markets, where data and publications serve as a valuable asset and the currency (Fecher et al., 2015), the pursuit of open science often feels like a philanthropic activity. As ECRs evolve from trainees to principal investigators (PIs), scientists, and professors, they navigate through identity and existential reflections concerning the impact and significance of their work. During this process, the accelerating pace of novel research publications concomitant with growing evidence on the reproducibility crisis is bound to invoke cognitive dissonance (Hensel, 2020).

At a personal level, this ECR career trajectory may accompany the five stages of scientific grief: (1) denial of the reproducibility crisis (*Problems*, 2007), (2) anger over assumed responsibility and possible culpability on reliability of published findings, (3) “bargaining” and barter of data and credit exchange (e.g., publications and funding), (4) despondency due to the glacially paced reform of the academic status quo, and (5) a hesitant and belated acceptance that the science without reproducibility and replication by the peers is not truly science (Wikipedia Contributors, 2024). The practical manifestation of these stages is driven by the available resources and metrics (personal and institutional) of successful scientific contributions. Here, we hope to address the modifiable, early risk factors of this grief and make a case for setting research priorities that invest in standards, tools, and training to enhance open and reproducible scientific methodologies. In this context, we introduce ECRU, an ECR, who embodies the dilemmas and decisions at the crossroads of traditional and open-science research lifecycle (seeBox 1), as the protagonist of our guide.

Box 1. **Case study—The journey of ECRU in open and reproducible neuroscience research**

*Background*: “ECRU,” an ECR in neuroscience, is keen to engage with open and reproducible science practices, but is hesitant to invest time to navigate the maze of standards, tools, platforms, and associated buzzwords. This case study describes ECRU’s guided tour from the initial steps of data curation to the final stages of publishing, illustrating the practical challenges and proactive solutions that simplify the adoption of best practices and improve the reliability of research.

*Step 1: Curation and Organization*

ECRU starts with a messy dataset comprising imaging and tabular data stored in a hard drive. ECRU wonders how many subjects with specific MRI sequence and neuropsychiatric assessment exist in there. Recognizing the need for structure, ECRU begins with a comprehensive manifest file to track data availability and employs the Brain Imaging Data Structure (BIDS) to organize data.

*Step 2: Processing and Analysis*

Understanding the importance of reproducible data processing over time and compute environments, ECRU opts for containerized pipelines (Docker or Apptainer) for data processing and analysis. ECRU adopts Git for managing and versioning code base and writes detailed documentation using MarkDown. Beyond text format, ECRU also creates Jupyter notebooks to share the analysis snippets and figures.

*Step 3: Annotation and Harmonization*

Anticipating the need for harmonization of variables across datasets for future studies, ECRU creates a data dictionary. In the data dictionary, ECRU annotates key variables’ “names” and “encoded values” in the analysis by providing descriptions and, when possible, citing a vocabulary or ontology standard to help re-use the data without having to contact original authors and replicate findings.

*Step 4: Publishing*

ECRU knows research objects include more than a manuscript. Thus, ECRU shares on data portals and platforms: (1) the manifest with an exact list of participants used in the analysis, (2) (if possible) source and processed data used in the analysis, (3) details of the containerized pipeline with well-documented code, (4) user-friendly documentation website and Jupyter notebooks, and (5) manuscript preprint for ensuring community access and feedback.

*Conclusion:*

ECRU’s journey in essence follows the FAIR principles, ensuring the research is findable, accessible, interoperable, and reusable. This deliberate effort enhances the credibility of ECRU’s research methodology and, by extension, the larger neuroscience research. In a nutshell, we hope that this case study serves as a relatable and practical guide to many ECRs navigating the open-science landscape and makes it easier for them to adopt these best practices.

ECRU is aware of the surge in neuroinformatics initiatives over the past two decades (seeFig. 1), aimed at (1) building consortia and data portals, (2) improving tools and platforms, and (3) expanding community standards and frameworks in service of open and reproducible science. Data-sharing projects such as LONI (Van Horn & Toga, 2009), INDI (Biswal et al., 2010), HCP (Van Essen et al., 2013), and OpenNeuro (Poldrack et al., 2013) heralded the era of high-powered neuroimaging studies. Simultaneously, platforms and tools such as NITRC (Kennedy et al., 2016) and NiPy (K. Gorgolewski et al., 2011) improved standardization and reproducibility of image processing pipelines. Availability and increasing adoption of the publishing platforms GitHub, Zenodo, and BioArxiv simplified sharing and open access of code, data, and scientific findings. Despite these significant efforts, the field is experiencing a major reproducibility, replicability crisis (Botvinik-Nezer et al., 2020;Glatard et al., 2015;Poldrack et al., 2017). In response, the more recent open-science efforts have increasingly focused on ensuring reliability and reproducibility of scientific findings through standardization of data curation and scientific reporting as well as of enabling end-to-end reproducible workflows. The FAIR principles (Wilkinson et al., 2016) were proposed and supported by the organization International Neuroinformatics Coordinating Facility (INCF) (Abrams et al., 2021), which translated into a landmark data standard of the Brain Imaging Data Structure (BIDS) (K. J. Gorgolewski et al., 2016). The standardized data organization enabled standardized processing giving rise to several BIDS apps (K. J. Gorgolewski et al., 2017) such as MRIQC (Esteban et al., 2017), fMRIPrep (Esteban et al., 2019), QSIPrep (Cieslak et al., 2021), BrainSuite (https://brainsuite.org/), and many more (https://bids-website.readthedocs.io/en/latest/tools/bids-apps.html). The concomitant development of container technologies (Kurtzer et al., 2017) further improved the portability of many processing tasks, thereby facilitating the replication of experiments and quantifying the effect of analytical flexibility. Additional large-scale initiatives have targeted key areas such as data management (e.g., DataLad), provenance tracking (e.g., Boutiques,Glatard et al., 2018), data reporting (e.g., COBIDAS,Nichols et al., 2017), collaborative analytics (e.g., COINSTAC,Ming et al., 2017), and workflow management (e.g., brainlife.io,Hayashi et al., 2024, FAIRly big,Wagner et al., 2022, NeuroDesk,Renton et al., 2024).

**Fig. 1:**
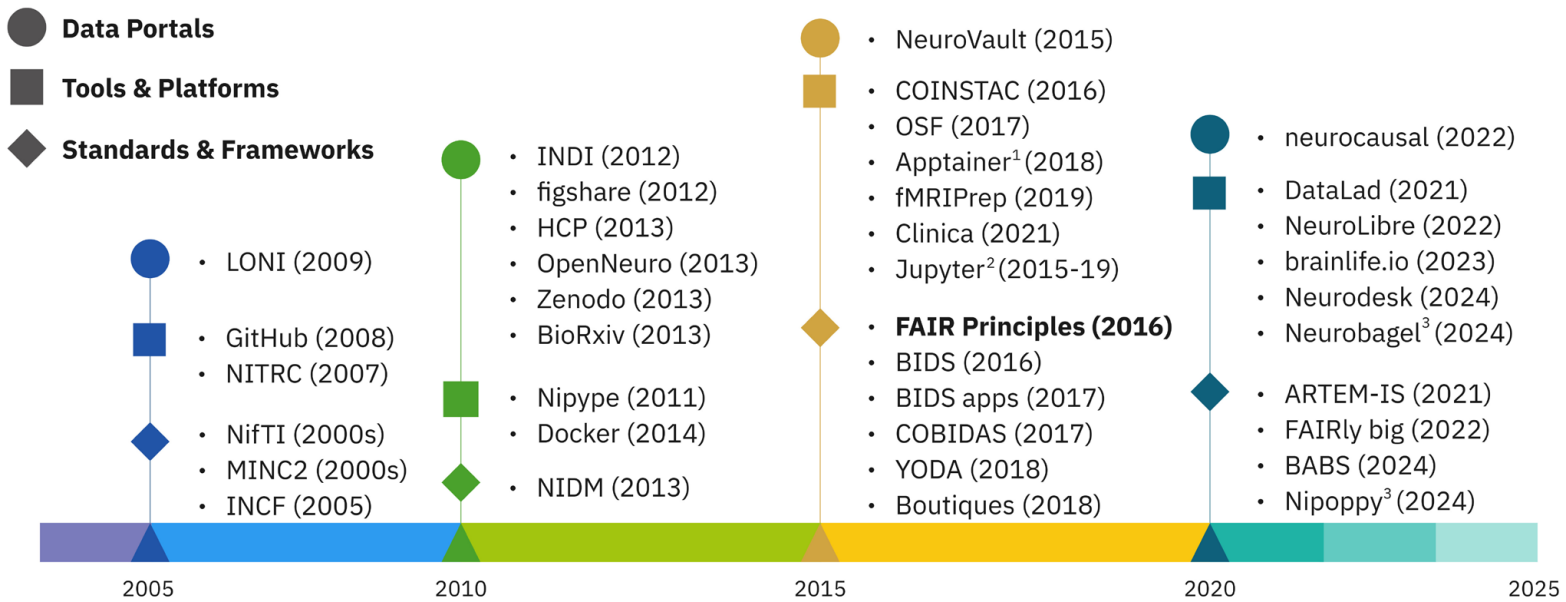
Initiatives over the past two decades in data sharing, development of standards, tools, and frameworks geared towards supporting open and reproducible science practices. The years are based on major publication or release dates. The FAIR principles are highlighted to acknowledge their motivating and guiding impact on this work. Notes: [1] Apptainer was previously known as Singularity. [2] Jupyter project has released several tools including JupyterBook, JupyterLab, and JupyterHub. [3] Disclosure: Authors N.B. and S.U. are active developers of Nipoppy and Neurobagel.

On the training side, ECRU has attended workshops and hackathons that are the cornerstones of Open-Science that bring about a cultural shift. Foundational efforts, such as the first Brainhack events (Cameron Craddock et al., 2016;Gau et al., 2021), laid the groundwork for a movement that continues to thrive. Recent initiatives, particularly organized by the Brainhack community, have played a crucial role in promoting open, inclusive, and community-driven neuroscience, developing numerous open-source tools and resources (e.g. YODA principles (Hanke et al., 2018), and Cobidas checklists (Nichols et al., 2017)) that are freely available to researchers (Gau et al., 2021).

So if we are progressing towards the right direction with growing awareness, tools, standards, and shared data—what exactly are we contributing to this*journal-entry*? The current challenge, as we see it, is effective navigation of this rich landscape of resources in practice—which often can lead into confusingrabbit holes* consuming the entirety of weekends. Here we thus aim to bridge the gap between the aspirational aims of FAIR principles (Wilkinson et al., 2016) and the adoption of appropriate standards, tools, and best practices for one without getting lost. We address common hands-on research challenges in the daily life of a trainee or an ECR and share a template protocol for proactive mitigation of these challenges and long-term peace of mind.

The scope of our guidelines covers the research timeline comprising data curation, processing, and publishing stages. This excludes the data acquisition phase. Within this timeline, we mainly focus on decisions related to (1) data types (e.g. MRI, EEG, clinical phenotypes), (2) compute setup (e.g. environments, containers), (3) metadata (documentation, annotations, provenance), and (4) research object publications (e.g. manuscripts, documentation, data). We only briefly discuss topics related to code and analysis as the relevant best practices have been well described previously (Balaban et al., 2021;K. J. Gorgolewski & Poldrack, 2016). We hope this provides a starting point for ECRs for the rapid adoption of open practices and reproducibility tools with minimal disruption and demand on their time and resources.

## 2 The Gap Analysis Survey

To identify current challenges in daily research tasks, we surveyed the neuroimaging community. Here are the summary findings of the survey (n=42). The complete results are provided in theSupplementary Materials. We note that the survey sample is relatively small and biased towards researchers interested in FAIR principles and neuroinformatic tools. Therefore, we expect the insights from the results would provide an optimistic estimation of the current challenges. We plan to keep this survey live and allow readers of this guide to submit their responses which we will make available publicly.

## 3 Our Vision and Aims for This Guide

With this article, at the cultural level, we seek to motivate trainees and ECRs to embrace the principles of open, reproducible research, and recalibrate the priorities of novelty versus reliability. On a practical front, we hope to provide a navigational compass, and a template workflow to help demystify data curation, processing, harmonization, and documentation, and publishing processes (seeFig. 2). Drawing from our own experiences and insights from an ECR survey, we discuss common challenges and scenarios, offering specific solutions we have adopted. We note that this is only a partial overview of current open tools and platforms. It is a reflection and recounting of lessons learned through our own journey as ECRs tasked to set up large-scale research pipelines and workflows in our laboratories and multisite collaborations. We narrate our experiences and advice on the aforementioned four steps with the following arc: we premise a familiar scenario, list specific decisions to be made, and provide our resolutions with key pain points, their remedies, and conclude with takeaways. We believe that these solutions can serve as a good starting point for new trainees and ECRs.

**Fig. 2:**
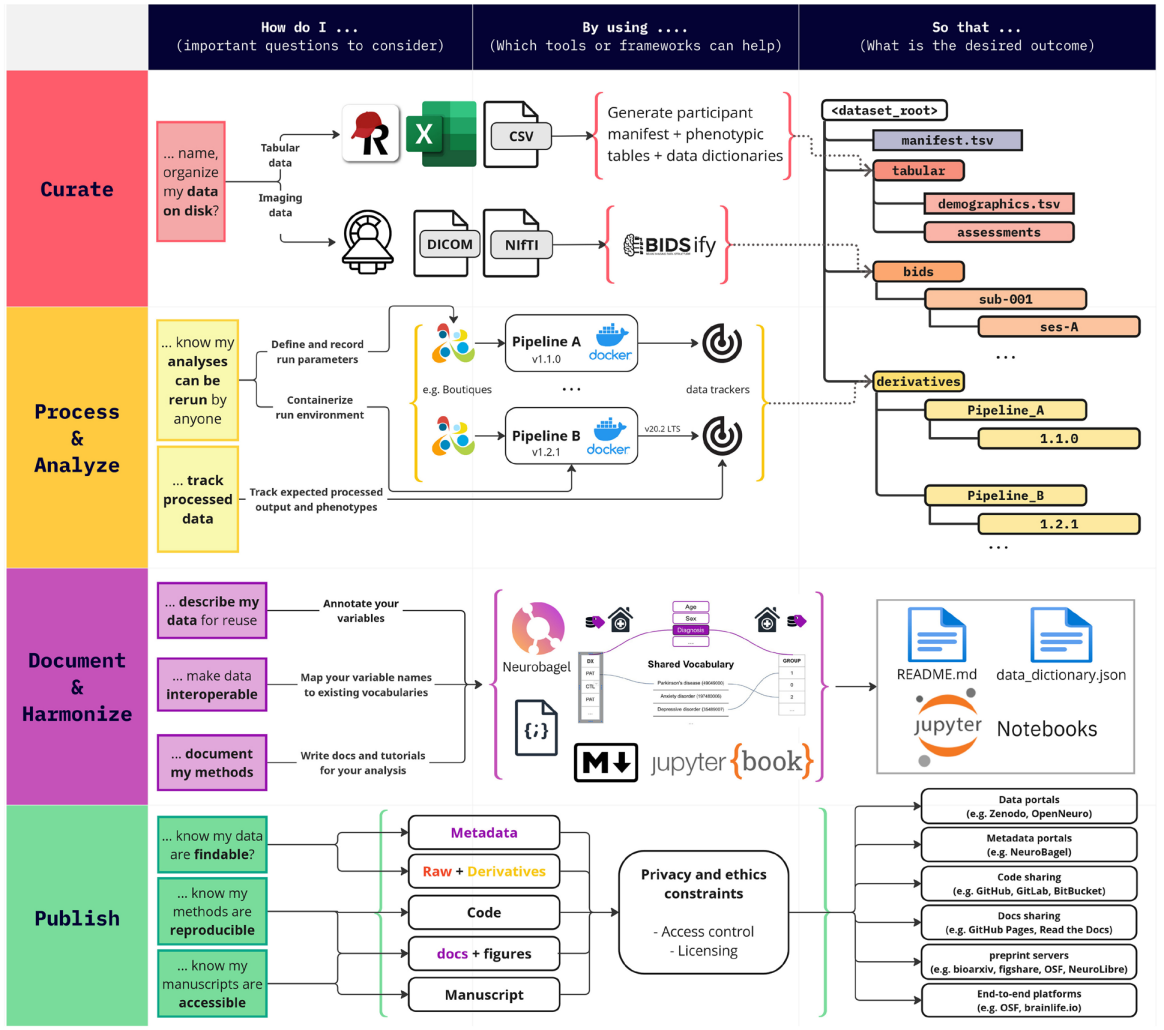
Overview of common tasks encountered in a neuroimaging research project once the data are collected. As we transition from trainees to ECRs, planning for all the stages from data curation to publishing findings becomes critical to proactively avoid pitfalls and ensure reliability of our research. The figure shows common questions encountered in this process and tools and standards that have helped the authors to ensure reproducibility, interoperability, and long-term sustainability of the projects. We note that this is not an exhaustive list of challenges or solutions, but a prototypical example of how to adopt FAIR research practices.

## 4 Step by-Step Guide for Aspirational ECR: ECRU

*As an early career researcher, postdoc, or data manager in neuroscience, you may find yourself grappling with messy data management followed by complicated data analysis. You are in the same boat as ECRU, dealing with a rich dataset full of brain imaging and clinical data. The excitement is palpable, but so is the weight of responsibility. It is challenging to ensure your work, particularly data and methods, is not only impactful but also credible. What challenges will ECRU encounter? How should ECRU plan and prepare for this long-term, multi-faceted process? We hope the step-by-step scenarios below will help guide the thought process as well as practical decision making.*

*Let us begin!*

Premise: There is a newly collected (or shared or downloaded) dataset with (supposedly) N subjects with imaging and tabular (demographic and clinical) data that need to be (1) processed, (2) analyzed, and (3) published ASAP.

## 5 Step 1: Curation

### 5.1 Scenario

ECRU is given a path to a directory on hard drive containing N subjects with ALL the data containing:

1. Large files: Raw imaging scans in a directory
2. Tabular files: Many spreadsheets and CSVs with messy human inputs (e.g. inconsistent date formats, missing and coded values without data dictionary—“999”, “-1”)

### 5.2 Likely reactions

Discombobulation from sheer number of files and folders and bewilderment to the naming choices.

### 5.3 Common questions

1. How many subjects are recruited?
2. Do we have information on multiple visits/follow-ups?
3. What data types from imaging and clinical assessments are available for each subject?
4. How should I name and organize imaging and tabular data?
5. How do I track availability, and new additions, and identify missing data?
6. How/when do I think about data versioning and releases?

### 5.4 Our solutions

#### 5.4.1 Q1–Q3: data availability

We start addressing these questions by first building a*manifest.*The*manifest*is a tabular file inspired by the “participants.tsv” file in the BIDS standard with a few key extensions and serves as the expected data availability record for a given study (seehttps://nipoppy.readthedocs.io/en/latest/). The manifest schema simplifies support for multimodal data (e.g., imaging, clinical, behaviour) collection especially in longitudinal designs. In a*manifest*each row represents a unique {participant x visit} record. The visits represent all data collection events including participant screening, medical questionnaires, clinical evaluations/tests, biofluid draws, and imaging acquisitions. Depending on the study protocol, visit naming can be standardized to follow ordinal (V0, V1, V2,…), or temporal (baseline, m12, m24,…), or purely categorical (preop, postop, clinical follow-up,…) convention. The other columns in the manifest can then denote the availability of tabular and imaging data types. We emphasize that the key procedural point here is to generate a record of expected sample sizes at the very beginning of data curation and processing using some standard. In certain study designs, BIDS “participants.tsv” or another standardized file format would also suffice.

##### 5.4.1.1 Potential pain points and remedies

Automating the manifest generation and update process can be highly dependent on data collection protocol. Although out of scope for this work, we recommend using digital data-capture software and databases with an Application Programming Interface (API) to facilitate automation and minimize human data entry errors.

##### 5.4.1.2 Takeaways

Let us not on-board ourdata-passengers* ontolong-haul data processing-flights* without the manifest verification!

#### 5.4.2 Q4: data naming and organization

For a given study, we address the organization and naming of data separately for (1) acquired (i.e., raw) imaging data, (2) processed (i.e., derived) imaging data, and (3) tabular (e.g., phenotypic) data. Our practice begins with creating a dataset directory tree for a single study (seeFig. 3). We then organize each of these data types into separate subdirectories within the dataset directory. For imaging data, we adhere to the BIDS standard, utilizing open tools, such as HeudiConv (Y. O. Halchenko et al., 2024), dcm2bids (Boré et al., 2023), and BIDSCoin (Zwiers et al., 2021) for MRI dataset, to convert and organize DICOMs into BIDS standard. Similarly, for EEG data, we employ BIDS-compliant converters such as EEG-BIDS tools (e.g., EEGLAB’s BIDS plugin or MNE-BIDS (Appelhoff et al., 2019)) that facilitate the structured organization of raw EEG recordings, ensuring adherence to standardized naming and file organization principles as specified by the BIDS EEG extensions (seeTable 1). The processed imaging data subdirectory is further organized by the specific processing pipeline and its version, maintaining flexibility in the structure of the actual derived outputs. The tabular data directory comprises a*demographics*file and*assessments*subdirectories to differentiate the universally available population descriptors (age, sex, etc.) from study-specific information. For all tabular data files within this directory, we recommend generating and maintaining comprehensive data dictionaries, ensuring clarity and uniformity in data interpretation (more details in Step 3). We recognize that BIDS provides guidelines and has extension proposals in progress for organizing derived and phenotypic data. We highly recommend engaging with and contributing to these community efforts to help build consensus.

**Fig. 3:**
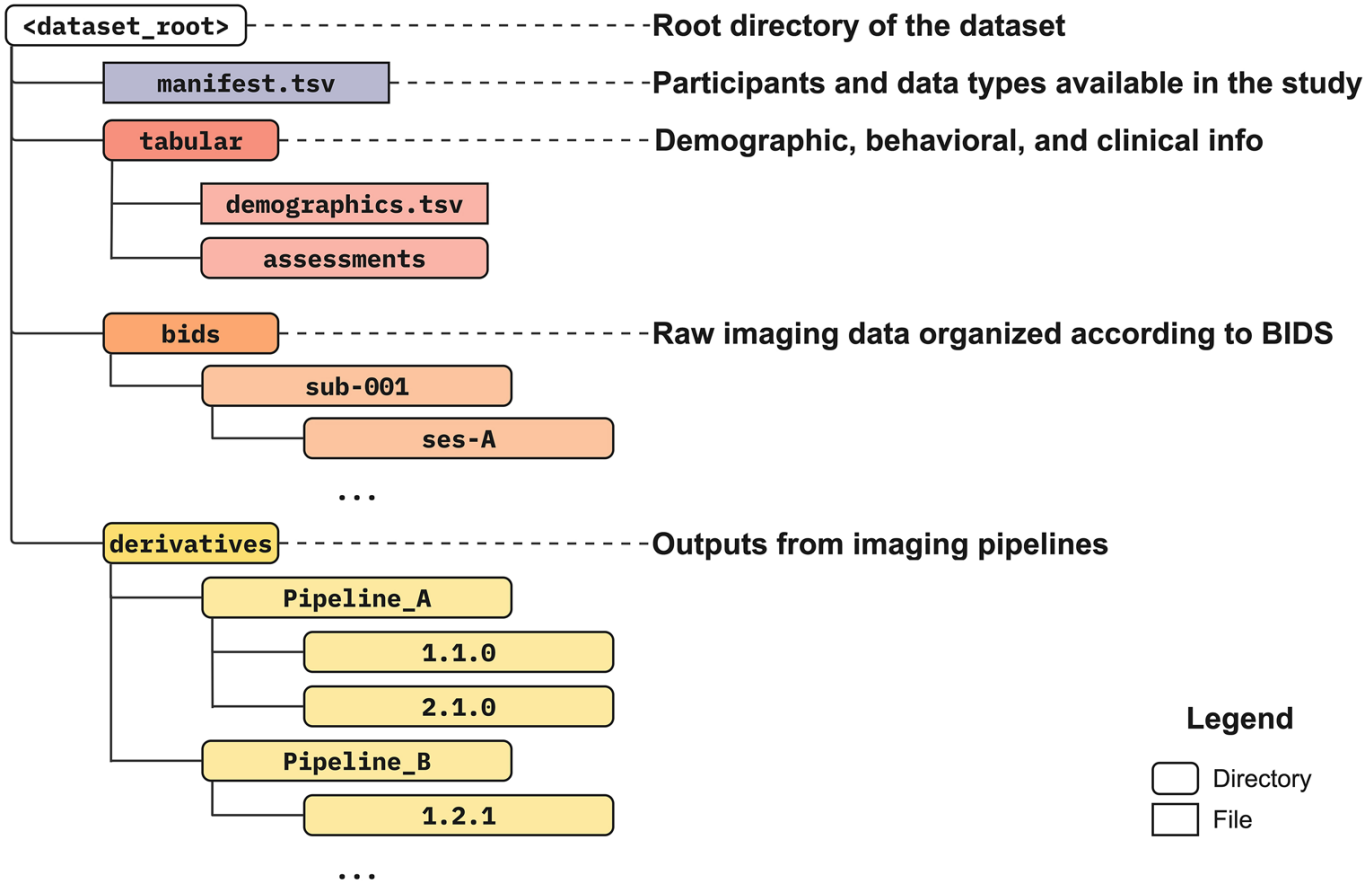
A suggested directory tree layout inspired by BIDS for a given dataset. The idea is to organize tabular, imaging (raw and processed) data with a schema that can be reused across datasets. The exact layout could be customized to fit one’s preferences. Nonetheless having “some” standard for organizing multiple linked data types and growing number of derivatives can help subsequent processing and harmonization tasks and simplify data discovery and reproducibility of your analysis.

**Table 1.** Overview of popular, open-source, active BIDSification tools that we have tried.

Tool	Input	Flexibility/learning curve	Technical skills	Comments
HeuDiConv ( Y. O. Halchenko et al., 2024 )	DICOMs	High Time: few weeks depending on MRI protocols	Intermediate Python	Relies on a custom heuristic.py file in python CLI
Dcm2Bids ( Boré et al., 2023 )	DICOMs	Medium Time: few days	JSON	Relies on a detailed config.json called from python CLI
BIDScoin ( Zwiers et al., 2021 )	DICOM, NiFTi EEG (plugin)	High Time: few weeks depending on MRI protocols	Low basic Python	Relies on a local GUI
ezBIDS ( Levitas et al., 2023 )	DICOM, NiFTi	Medium Time: few days	Use of browser	Relies on a web GUI Requires data upload on ezBIDS server.
MNE-BIDS ( Appelhoff et al., 2019 )	EEG	Medium Time: few days	Basic python	Offers both GUI and script support

For a detailed list see:https://bids.neuroimaging.io/benefits. The estimates of flexibility and learning curve are based on our experience from organizing hackathons/workshops and primarily for the multi-protocol (i.e., structural, diffusion, functional) studies with availability of DICOMs. Individual experience may vary based on familiarity and technical preferences.

For EEG data, adherence to the latest BIDS EEG extensions is recommended to ensure that data organization aligns with community-accepted standards, enhancing the interoperability and reusability of the data. This approach not only facilitates consistency across different imaging modalities but also promotes a standardized methodology for data management and sharing within the neuroscience research community.

##### 5.4.2.1 Potential pain points and remedies

BIDS conversion can be messy and often requires manual specification of tedious heuristic mapping of scanner protocols. Although out-of-scope for this work, we highly recommend adopting standardized naming of protocols, such as ReproIN (Reproin: A Setup for Automatic Generation of Shareable, Version-Controlled BIDS Datasets from MR Scanners, n.d.).

##### 5.4.2.2 Takeaways

Although it seems like a simple directory and file naming exercise, this is a deceptively difficult task highly susceptible to human errors and*NaM1ng_eCcEntriCit1es*. Be mindful, given this will serve as the origin story for all your analysis and high-impact publishing adventures, it is worth investing a significant amount of time to avoid downstream disasters and ensure reproducibility of your findings.

#### 5.4.3 Q5: data tracking—reality on the ground

As mentioned earlier, we use*manifest*as the expected availability of all participants and collected data types. This simplifies verification of available raw imaging files and tabular files. Particularly BIDSified datasets can be easily queried and validated using the pybids API. Tallying availability counts for the processed imaging data is trickier since it is contingent upon successful completion of associated pipelines that generate the customized outputs. We address this problem by writing configurable “trackers” (seehttps://nipoppy.readthedocs.io/en/latest) to check for a list of minimally expected output files for a given pipeline cross-referencing against the*manifest*. These trackers help detect missing data and processing failures. We have also built light-weight, interactive web dashboards to visualize this tracking information (seehttps://digest.neurobagel.org/). These user-friendly interfaces have turned out to be a huge time saver by catching unintentional data drops and facilitating quick relaunching of failed processing.

##### 5.4.3.1 Potential pain points and remedies

Data tracking can get tricky if your study is ongoing (i.e. prospective) compared with completed (i.e. retrospective) ones. In these situations, maintaining proper sequence of*manifest*updates and subsequent processing is crucial. If your dataset consists of multiple imaging visits (i.e. bids sessions), we recommend processing each visit separately, unless a pipeline expects or benefits from longitudinal input. This is primarily to avoid inconsistent input to pipelines some of which may average certain scans across sessions. If using pybids-dependent BIDS-apps, this can also help use of session-specific filters to accelerate indexing, archiving, and simplify job submission on a compute cluster.

##### 5.4.3.2 Takeaways

Data are a valuable asset. Let us make sure we do not have aleaky wallet*.

#### 5.4.4 Q6: data versioning and releases

Although we tend to think of collected data as static objects, in practice that is rarely the case. We encounter questions related to versioning and releases in two common situations. First, especially in prospective studies, data***—***just like your codebase***—***are naturally growing, getting updated, and rectified when mistakes are noticed. We recommend using flexible, open-source tools such as DataLad (Y. Halchenko et al., 2021) to help you track and version your data. Second, depending on the research question, a different slice of data (aka view) needs to be filtered. In both of these cases, it is super useful to have a unique digital object identifier (DOI) or Research Resource Identifiers (RRID) associated with a given data slice-time epoch. These identifiers can be used for internal versioning or wider releases with collaborators or open databases. Several open tools and public digital object repositories (e.g., OSF, Zenodo) are available to assist you with versioning and minting DOIs and RRIDs.

##### 5.4.4.1 Potential pain points and remedies

Unlike code, data objects are heavy and require a considerable amount of resources and expertise to handle versioning and releases. For large studies, we recommend hiring dedicated data managers for these tasks. When permitted by the data governance policy, we highly encourage uploading well-curated data-slice used in your analysis to open repositories and sharing the release tag in your publications.

##### 5.4.4.2.Takeaway

Imagine a world where you do not have toplay 20 questions*with your colleagues, your PI, your collaborators, or the authors from your favorite paper to guess the exact dataset they used.

## 6 Step 2: Processing & Analysis

### 6.1 Scenario

ECRU is asked to start a project involving:

1. A shiny new (pre) processing pipeline with a recent buzz
2. Three different statistical analyses

### 6.2 Reactions

Stress from figuring out software installation; indecision on configuring pipelines parameters; overwhelm from managing computing cluster setups.

### 6.3 Common questions

1. What is the preferred way of installing and setting up software?
2. How do I ensure I am running the pipeline with appropriate parameters?
3. What do I do when certain participants fail to process?
4. How do we handle quality assessments?
5. How do I set up a code environment for my analysis?
6. How do I ensure reproducibility of my analysis?

### 6.4 Our solutions

#### 6.4.1 Q1–2: data processing setup

Installation of neuroimaging processing software or toolboxes can be a daunting task riddled with unexpected issues related to OS, dependency tree conflicts, proprietary software licenses, cluster deployments, and many more. Fortunately, in the community, there is a growing availability of ready-to-use containers for popular pipelines. Thus, when available, we highly recommend use of Apptainer (formerly known as Singularity) or Docker containers for processing data locally or on compute clusters (i.e., HPCs or cloud infrastructures) as they ensure reproducibility and proper versioning.

Neuroimaging pipelines typically offer a highly flexible configuration of input parameters which depends on the data acquisition protocol and quality (see (Botvinik-Nezer et al., 2019;Clayson et al., 2021;Šoškić et al., 2022;Trübutschek et al., 2024). Unless documented in great detail, it is often impossible to know the “correct” set of parameters used by original authors or collaborators, complicating the replication or comparison of analysis. To avoid such scenarios, we recommend the adoption of tools such as Boutiques (Glatard et al., 2018) that can automate reproducible execution and provenance of processing. Alternatively, one can manually generate, version, and share descriptive run scripts and run-time parameter config files (e.g., JSONs) that keep the record of the executed command with all the relevant input parameters.

In contrast with MRI modalities, EEG data processing poses distinct challenges stemming from the diversity of data formats, graphical user interface (GUI), and the lack of widely adopted preprocessing standards. While tools such as EEGLAB (Delorme & Makeig, 2004), FieldTrip (Oostenveld et al., 2011), and ERPLAB (Lopez-Calderon & Luck, 2014) Studio (seeTable 2) provide flexible and accessible options, achieving consensus on standardized workflows remains an ongoing effort within the community. Thus, much more complex challenges persist in the EEG domain, underscoring the need for continued collaboration and innovation. Discussion of these challenges, mental toll, and possible therapeutic toolkit is out of the scope of this journal entry. Here, we provide a concise list of EEG tools and their intended use case inTable 2for the newcomers as a starting point. Similar decision challenges exist for GUI-based MRI software tools as well. We have included summary comparisons of these tools in the Supplementary Materials (seeTable S1).

**Table 2.** EEG GUI-based software comparison.

Software	Description	Best for
EEGLAB ( Delorme & Makeig, 2004 )	Open-source MATLAB toolbox for processing EEG/MEG data. Offers a GUI for comprehensive data analysis. Includes extensions such as ERPLAB Studio for event-related potential (ERP) analysis.	Comprehensive electrophysiological data analyses.
BrainVision Analyzer ( Brain Products GmbH, 2019 )	Commercial platform with a user-friendly intuitive GUI for a wide range of EEG data analysis tasks.	Teaching, non-programmers, and clinical EEG analysis.
FieldTrip ( Oostenveld et al., 2011 )	MATLAB toolbox with GUI functionalities for MEG, EEG data analysis, focusing on complex analysis tasks.	Combining GUI and script-based complex analyses.
MNE-Python ( Larson et al., 2024 )	Python package for MEG and EEG data processing, featuring GUI components for data exploration.	Script-based analysis with GUI data inspection capabilities.
BESA Research ( Iordanov et al., 2018 )	Commercial software for EEG and MEG data analysis with a comprehensive GUI.	Advanced EEG and MEG analysis, including source localization.
Brainstorm ( Tadel et al., 2011 )	Open-source GUI for EEG and MEG data visualization and analysis, supporting preprocessing to source estimation.	Integrated EEG and MEG data processing.
NeuroScan	Commercial software suite with a GUI for comprehensive EEG/MEG data acquisition and analysis.	Complete workflow for EEG/MEG data, from acquisition to analysis.

##### 6.4.1.1 Potential pain points and remedies

New shiny tools and pipelines pop-up every day. Which tools to select and adopt is a tricky question. We believe that evaluating the underlying principles and dependencies (e.g., community standards, modularity, open source) of the new tools can help with these decisions. Moreover, assessing documentation and maintenance support is also critical since sustaining software is much harder than creating it.

##### 6.4.1.2 Takeaways

Let us not depend on our fading episodic memory to remember exact CLI string or GUI screenshot to rerun or share our analysis steps.

#### 6.4.2 Q3: data processing troubleshooting

Image processing failures can happen for a multitude of reasons which we broadly separate into two categories. A pipeline can fail due to mismatched or insufficient compute resource allocations such as wall-time and memory. These are relatively easy to fix by simply relaunching the processing for the failed participants. In practice, however, this can easily become an annoyingly tedious task of parsing logs, cleaning-up intermediate files, and compiling job lists. This is where one can thank their past self if they successfully organized the data in a standard manner and employed “trackers” (seehttps://nipoppy.readthedocs.io/en/latest) to automatically flag failed participants. The second category of failures can result from data-related issues. For MRI, these might include registration or segmentation failures due to acquisition specific (e.g. motion) or biological image artefacts (e.g. atrophy). For EEG data, common issues include signal noise, electrode disconnections, or software-specific errors during preprocessing steps such as artefact rejection or signal filtering. These participants are either discarded or re-run with pipeline parameter finetuning. In these scenarios, the provenance of these custom runs becomes important and should be documented—ideally with the aforementioned open tools (e.g., Boutiques).

##### 6.4.2.1 Potential pain points and remedies

The time and effort needed to troubleshoot and maintain datasets can grow exponentially with the sample size and number of pipelines. Even a simple automated tracking script and a visual dashboard to tally successes and flag failures can go a long way to preserve your sanity.

##### 6.4.2.2 Takeaways

Let us minimize manual usage of CTRL-F, grep <participant_id>, clicking open unending list of folders, and prayers to the computational deities to ensure successful processing status.

#### 6.4.3 Q4: data quality assessment

The quality control (QC) and/or quality assessments (QA) for both imaging and tabular data are highly critical for ensuring accuracy of findings, and yet probably it is the most subjective and tedious of the tasks. Several tools such as MRIQC (Esteban et al., 2017) and VisualQC (Raamana, 2023) exist for automatic QC and also for manually rating the quality of raw and processed images looking for artefacts, distortions, or other issues that may affect the analysis, seeTable 3. Several processing pipelines, such as fmriprep and qsiprep, provide visual, browser-based QC reports on their processed output to the users. For EEG data, similar tools are available, such as EEGLAB (Delorme & Makeig, 2004) (provides various plugins and built-in functions), MNE-Python (Appelhoff et al., 2019) (offers functions for automated detection of bad channels), FieldTrip (Oostenveld et al., 2011) (includes tools for automatic artefact detection and manual inspection of EEG data), and Brainstorm (Tadel et al., 2011) (provides comprehensive tools for visual inspection and automatic artefact detection). Despite the availability of these helper QC tools, one still has to make decisions related to acceptable cutoffs, make exceptions, or manually verify and rate a set of images that depend on the research question and expert domain knowledge. Unsurprisingly, this often becomes a subjective and iterative process with differential QC criteria yielding overlapping but non-identical subsets of participants sampled from the source dataset employed towards an analysis. This poses an intractable challenge towards “standardizing” QC protocol that can work across datasets, modalities, or specific imaging-derived phenotypes (IDPs). Nonetheless, these tools and some human-in-the-loop QC protocol help filter out abject scanning or processing failures. This is critical since often pipelines will produce reasonable looking IDPs from completely failed scans. Thus, proper documentation of quantified ratings and accompanying notes needs to be generated and shared alongside every research analysis.

**Table 3.** Overview of tools for quality control.

Tool/approach	Description	Use case
MRIQC ( Esteban et al., 2017 )	Automated QC for MRI data, generating reports to identify problematic scans.	Pre-processing quality control.
VisualQC ( Raamana, 2023 )	GUI for manual inspection of neuroimaging data.	Manual quality control and inspection.
mrQA ( mrQA: Automatic Protocol Compliance Checks on MR Datasets — mrQA 0.2.2 Documentation, n.d .)	Automatic protocol compliance checks on MR datasets	Evaluate that MR scans are acquired according to the pre-defined protocol and to minimize errors in acquisition process
fmriprep ( Esteban et al., 2019 ), qsiprep ( Cieslak et al., 2021 ), Conn toolbox ( Whitfield-Gabrieli & Nieto-Castanon, 2012 )	Generates visual reports for QC of the processed data	Checking function and diffusion processing output
EEGLAB ( Delorme & Makeig, 2004 ), Brainstorm ( Tadel et al., 2011 )	Comprehensive plugins and visual interface for EEG QC	Visual inspection and artefact detection in EEG data
OpenRefine ( Delpeuch et al., 2024 )	Tool for tabular data cleaning	Ensure naming and encoding consistency in long tabular files

The approach for tabular data which encompasses demographics and clinical assessments requires a different quality control approach. Here, the focus is on data cleanliness, accuracy, and completeness. Validation scripts are employed to check for inconsistencies, missing data, or incorrect entries. This process ensures that all tabular data are accurate and ready for analysis. This can be achieved with the aforementioned*manifest*and a data-dictionary listing, at minimum, all the demographic and phenotypic variables, their data types (e.g., categorical, continuous), and valid range of values (e.g., 0–30).

##### 6.4.3.1 Potential pain points and remedies

The sheer volume and variety of data can make quality control a challenging task. Keeping track of different quality metrics for various types of data requires meticulous attention to detail. Quality control is not easy to standardize, and can be variable across raters and across projects. Employing automatic QC tools at-scale supplemented by time-permitting manual QC effort can be an acceptable strategy in many cases. When possible, crowdsourcing some of the manual effort and sharing the QC rating can be highly useful.

##### 6.4.3.2 Takeaways

In summary, data QC is a multi-faceted process that requires both automation and manual intervention. By establishing a protocol that makes sense for your data, one can improve the efficiency of monitoring and validating the quality of imaging and tabular data.

#### 6.4.4 Q5-6: reproducible statistical analysis

The statistical analysis phase is where output from the data processing pipelines translates into meaningful insights supported by statistical findings and figures. Just as containers support standardization and reproducibility in data processing tasks, they are equally valuable in encapsulating the full analysis environment, ensuring portability of your statistical workflows across systems and over time. However, in contrast to semi-standardized and largely sequential image (pre)processing tasks, analysis steps inherently tend to be more exploratory and iterative in nature as they are meant to make novel contributions. This necessitates a flexible setup to handle a growing codebase along with its dependencies, documentation, and links with the published results. Here we outline a TODO checklist of best practices to set up and perform a successful analysis in a scalable, reproducible manner.

1. Code versioning: Before you even write the first line of a code, create a git repository using your preferred tool, that is, Github, Gitlab, and Bitbucket. You can keep it private if you like, but creating it proactively, that is, before starting to code is critical rather than dumping your code retrospectively just before publishing.
2. Code tests and reviews: Software testing is an entire topic of itself which often remains an afterthought in the academic setting. To start, we recommend simply writing a handful unit tests and sanity checks for your analysis code. Then as a next step, you can add these tests into the online version control tools (eg. Github) to automate the testing. We also strongly recommend using these version control tools for code review which can play a huge role in the quality and efficiency of your code.
3. Create a README: Think of README as meeting notes from your inner monologue during a coding session. This is a live, potentially unstructured documentation to keep track of your thought process as you code. You should also version control this file in the git repo you have created. This can grow and mature in parallel with your codebase and can be reformatted with a styling template at the later stages.
4. Set up a virtual environment (or a container)—Creating a separate virtual environment for each of your projects is important as it can avoid messy setup issues and conflicts between underlying dependencies for your codebase and consequent headaches. If you are only using python for analysis, python virtual environments typically suffice this purpose and are lightweight on disc. Conda is a more versatile solution that not only offers an isolated environment but also provides package management. Conda also handles non-python dependencies and is more popular within the cross-platform, data science community. When starting, picking either of the two is fine, as long as you stick to one for a given project.
5. Code with care—As non-computer scientists or software developers, writing code often feels intimidating. And rightfully so. For the interested, there are loads of resources for learning, improving, and finessing your coding skills, but here we only mention a handful of tips in the context of analysis. First, especially as we are in the era of chatty, Large Language Model (LLM) powered coding bots that feast on your published code, separating your personal “config” from your code is critical. Refrain from hard coded directory paths, credentials inside your scripts even as comments. Instead save them as a local config file outside of your published code which helps with privacy as well as portability. Second, try to follow best practices for analysis design, reporting, and sharing as outlined inNichols et al. (2017),Pernet et al. (2018), andWiebels et al. (2019), within your code to help standardization and comparison or analytic approaches in neuroimaging. Third (which builds on top of the first two), modularize your code to support assessment of analytic “vibrations” or “flexibility.” This involves your ability to test the robustness of statistical findings against methodological variations such as choice of preprocessing software, versions, QC criteria, signal thresholds, and model selection. A swapable “config” file can really speed up these analytic iterations.
6. Document—when it comes to code docs, READMEs are the bare minimum self-help notes. As the codebase matures, one should aim for detailed doc strings, in-line comments, and comprehensive tutorials that could be published for other users. We discuss this later in the documentation section.

##### 6.4.4.1 Potential pain points and remedies

- Translating hypothesized analysis into efficient code is hard. Reverse translating someone else’s code into a logical thought experiment is even harder. Let us avoid getting lost in translations by putting deliberate effort into well-structured documentation and code reviews. We hope that coder LLMs can help with this process and save us a lot of time.
- Given the degrees of freedom in neuroimaging analysis, it is rather easy to take unintentionalstatistical random walks* to conclusions without rigorous validations. Writing your code centered around portability can greatly help crowdsource the validation process and improve FAIRness of your analysis.

##### 6.4.4.2 Takeaways

Statistical analysis is an exciting area of exploration, discovery, that is muddled with traps of p-hacking, double-dipping, and many more. Let us ensure the reliability of our analysis before celebrating the novelty of findings.

## 7 Step 3: Documentation, Annotation, and Harmonization

### 7.1 Scenario

ECRU is now asked to help

1. A new student or a laboratory member who also wants to work with the same data.
2. A collaborator who is asking for the raw imaging and tabular data along with information on processing steps.

### 7.2 Reactions

Confusion about level of detail, frustration from lack of common jargon

### 7.3 Common questions

1. How do I document my process so far? How do I create and organize READMEs?
2. How do I annotate my data and create data dictionaries?
3. How do I harmonize naming conventions (e.g. column names).
4. How do I share the details on my processing steps and QC?

### 7.4 Our solutions

#### 7.4.1 Q1: documentation

README files and laboratory notebooks are useful and typical ways to document for personal recollection and inform your colleagues. However, they often seem cryptic when read a few months in the future especially by someone other than you. It is, therefore, important to apply similar “FAIR” practices to your documentation that you would do to your data and code. That is, they should be organized in a way that is easy to find and accessed. And they should follow some standardization and be made available to the community for comments, issues, and updates.

Moreover, scientific documentation is more than writing text files. Good “how-to” guidelines should include figures and code snippets. The effort for formatting and packaging such multi-data-type content can be simplified with several open-source tools currently available. The best practices and extensive community efforts in this domain can be explored here:https://www.writethedocs.org/.

##### 7.4.1.1 Practical implementation

A standard documentation process involves three steps: (1) write, (2) build, (3) host. Writing can take the form of text files, figures, or doc-strings for your code. Subsequently to share the created content, it is built and served as web pages with a user-friendly interface. Finally, these web pages are hosted on a website (free or paid) to be accessible publicly. Several tools exist to simplify this process and to maintain and update documentation over time. For a detailed primer on these tools and their specific uses, refer to this guide (Gau, n.d.), which outlines effort levels for converting simple README files into well-formatted documentation pages. Below is a brief overview of these tools, commonly used to document research pipelines (seeTable 4). While these tools are often associated with code documentation, they are equally effective for documenting data handling, processing steps, and metadata, especially when code and data workflows are integrated.

**Table 4.** A brief overview of selected tools (and their combinations) commonly used for documenting research pipelines, including both code and data workflows.

Write	Build	Host	Purpose	Effort needed	Advantages	Examples
Markdown	Jekyll (behind the scenes)	GitHub Pages	Basic (e.g. README) instructions and notes for your analysis	Little	No new installation or setup required.	GitHub Pages examples
Markdown	Jekyll	GitHub Pages	Make personal websites and serve basic documentation for a simple project	Little	Easy setup, many extensions and plugins for pretty UI layouts	Many personal websites of researchers
Markdown	Jupyter Books	GitHub Pages	Serve documentation + code jointly	Little	Good for tutorials	Sci-kit learn MOOC
Markdown + MyST	Jupyter Books	Netlify	Serve markdown + code jointly with greater interoperability	Medium	Good for tutorials. MyST can support many publishing formats	The Turing way
Markdown	Jekyll / Hugo	Netlify	Render official looking project documentation website	Medium	Allows quick preview of web pages before deployment	OHBM Open-Science SIG
Markdown	MkDocs	Read the docs	Render official looking project documentation website	High	Easy local setup and integration with github code	BIDS Specs
Markdown + Python doc strings	Sphinx	Read the docs	Create a documentation website directly from python code base	High	- Renders doc strings directly from python code - Support other languages (e.g Matlab/Octave) - Simplifies and syncs versioning	Nipoppy (… and pretty much the docs website of any python package)

While these tools are often associated with code documentation, they are also useful for documenting data handling, processing steps, and metadata, especially when code and data are intertwined in the research process. In our experience, effort levels range from little (few hours, no coding background) to medium (couple of days, basic Python knowledge), and high (couple of weeks, intermediate Python proficiency).

##### 7.4.1.2 Potential pain points and remedies

Documentation is time consuming and admittedly not the most fun part of research. Good documentation, whether for code or data, requires honed communication skills to express your work with clarity and detail to the readership with a wide range of expectations and skill levels. Nonetheless, in the long run, it actually saves time, helps catch errors, and even refines your own thought process. To make this process less painful, we highly recommend scheduling weekly group documentation sessions similar to laboratory meetings or journal clubs.

##### 7.4.1.3 Takeaways

The best way to truly understand a topic is by teaching it to someone else!

#### 7.4.2 Q2: data dictionaries—annotating data for humans

Data annotation and semantic harmonization primarily deal with tabular data files comprising demographic and phenotypic information of our research participants. We need to annotate these data because unlike standards such as BIDS for imaging data, with tabular data we are largely on our own in how we name columns and encode values. When we annotate data, we have two types of audiences in mind: other human researchers (including ourselves in 6 months) and computers who need to do something with our data. To annotate and describe data, we create a data dictionary. A data dictionary should describe each column in a tabular file, explain what kind of information is stored in it, what the values in the column mean, and so on. Although this information can be written in any text file, it is important to use machine-readable formats such as JSON or YAML—even if the file is intended only for other humans—because the clear structure of such a file is much easier to read and work with.

Here is an example data dictionary (participants.json) that describes your variables

**Table:** 

{ “age”: { “Description”: “age of the participant”, “Units”: “years” }, “sex”: { “Description”: “sex of the participant as reported by the participant”, “Levels”: { “M”: “male”, “F”: “female ” } }, “group”: { “Description”: “group variable”, “Levels”: { “PD”: “Parkinson’s patient”, “CTRL”: “Control subject”, }, }

##### 7.4.2.1 Potential pain points and remedies

Lack of community standard inevitably creates subjective variable names and ontologies. Thus, it becomes a time-consuming task to describe your set of variables in sufficient detail especially if you have a large number of measurements/assessments. On a positive side, several projects (e.g. BIDS Pheno (https://bids-phenotype.readthedocs.io/en/latest/), NeuroCausal (https://neurocausal.github.io/), COBIDAS (Wiebels et al., 2019), Neurobagel (https://neurobagel.org/), and phenopackets (Jacobsen et al., 2022)) are gathering momentum that would help the community standardize, annotate, and share their data dictionaries and related metadata. For EEG data, the BIDS-EEG extension provides a standardized framework for organizing and sharing EEG data, ensuring consistency and interoperability across studies.

##### 7.4.2.2 Takeaways

An ambiguous column name or value encoding can cause downstream disasters. It is always wise to prepare and check data dictionaries to ensure your exciting significant results are not caused by creatively encoded (e.g., 999) missing values!

#### 7.4.3 Q3: harmonization—annotating data for machines

A complete data dictionary already is a massive help to understand and work with tabular data, especially if you share your data with others or you reuse data from other people. However, often we do nt work on just one dataset but instead need to understand and combine several datasets into one. Even if we know from the data dictionary that the “DX_GROUP” column in one dataset and the “diag” column in the other dataset both describe the clinical diagnosis of a participant, or that a value of “1” in the first column, and “PD” in the second column both mean that a participant had a diagnosis of “Parkinson’s disease”, to combine both datasets we need to align their naming and format, that is, harmonize them. We could pick the particular format of one dataset and align all others to it, but this quickly becomes a very manual and tedious process with many exceptions, for example, if our “reference” dataset does not include all variables in the other datasets.

A much better solution is to align each dataset individually with an existing standard for naming information that is also used by other researchers. These standards are called “controlled vocabularies” or “taxonomies” and are curated lists of terms with clear definitions that we can use to be precise about what our data mean. Often, they come from ontologies that are general descriptions of a domain. The terms in these lists also come with unique numeric identifiers so machines can easily understand and process them. An example of a “controlled vocabulary” is the International Classification of Diseases (ICD) that is curated by the World Health Organization to allow doctors and researchers to use unambiguous terms when referring to clinical states (World Health Organization, 2018). We could replace the ambiguous values for “Parkinson’s disease” in our datasets with “8A00.0” the unambiguous numeric identifier for “Parkinson’s disease” from the ICD-11 to harmonize them. But changing the raw data in our tables is often not a good idea, and numeric codes are also not very readable. The best solution is, therefore, to leave the “1’s” and “PD’s” in their tables and instead add the numeric code for the controlled term to the data dictionary where a computer can understand and process them.

##### 7.4.3.1 Potential pain points and remedies

Finding good, controlled vocabularies and terms for variables, and writing machine-readable data dictionaries need a lot of technical knowledge about things that are not very important for our research. So it is a good idea to rely on software to make this easier. Projects such as Neurobagel and OpenRefine can help with this task by providing user-friendly interfaces to annotate your existing data with FAIR vocabularies.

##### 7.4.3.2 Takeaways

Harmony within the study variables is music to ears for a researcher working with multiple datasets!

## 8 Step 4: Publishing Research Objects

### 8.1 Scenario

ECRU needs to submit the manuscript, code, and potentially analysis-ready dataset.

### 8.2 Reactions

Exhaustion, trepidation for reviewer 2’s insatiable desire to ask for major revisions.

### 8.3 Common questions

1. What research objects do I share along with my manuscript?
2. How and where do I share my data? Do I have the right? What about privacy?
3. How do I share my code and documentation? What about licensing, data usage agreements?
4. Where do I submit my manuscripts? What about preprints?

### 8.4 Our solutions

#### 8.4.1 Q1: sharing a workflow with multiple research objects

A published manuscript is a much-desired outcome and is considered to be a benchmark of successful research contribution. Nonetheless a standalone manuscript seldom contains sufficient detail to safely reuse or fully understand the reported experimental findings. Hence in recent times, there has been a push for publishing research objects beyond a PDF. These may contain data (raw or processed), code, figures, documentation, environments, and anything else that could assist in reproduction and extension of the experiment. Several portals and platforms exist for publishing these research objects either separately or jointly—a choice that depends on the scale and privacy constraints of the research. The space of general-purpose data publishers (e.g., object stores) is quite large and beyond the scope. Here we mainly focus on solutions for publishing light-weight datasets accompanying analysis and figures in a manuscript. SeeTable 5for brief comparison of these platforms.

**Table 5.** A brief overview of data publishing platforms.

Platform	Purpose	Support for (data/code/compute)	Pros	Cons	When to use it
Brainlife ( Hayashi et al., 2024 )	Neuroscience platform for processing, analyzing, and visualizing brain data	data/code/compute	Comprehensive ecosystem for neuroimaging supports various data formats including BIDS, integrates with processing pipelines	Requires learning curve to use platform effectively, limited to brain data	To share and analyze brain imaging datasets, especially useful for processing and visualization tasks
Zenodo ( European Organization For Nuclear Research & OpenAIRE, 2013 )	General purpose platform for data and code	data/code	Simple process, allows for access-restricted data, run by CERN	50 GB limit. Search is limited to keywords	To share data and code in the same place
OpenNeuro ( Markiewicz et al., 2021 )	Neuroscience platform for openly accessible BIDS data	data	Well-curated, domain-specific platform, validates data	No solution for sensitive data or data that cannot be stored on US servers	To share a BIDS dataset with the widest possible audience
NITRC ( Kennedy et al., 2016 )	Neuroimaging platform for data sharing and compute	data/code/compute	NIH-supported resource meeting stringent FAIR sharing and open-access requirements	Outdated user interface	Browse, search, and compare open projects, datasets, and software
NeuroLibre ( Karakuzu et al., 2022 )	Neuroscience publishing platform for executable articles (data + code)	data/code/compute	Domain-specific promise to keep interactive articles running	Submission process can be technical and require extra work	To share your literate programming research reports in an easily accessible way
OSF ( Foster & Deardorff, 2017 )	Generic data publishing platform	data/code			
NeuroVault ( K. J. Gorgolewski et al., 2015 )	Neuroscience metascience platform for statistical effect maps	data	Good place to share statistical maps with keywords	Not for sharing entire dataset	When you publish your data on a dedicated platform, publish your statistical maps on neurovault

Seehttps://f1000research.com/for-authors/data-guidelinesfor more in-depth review and guidelines.

To facilitate the joint publication of multimodal research objects, open platforms such as NITIC (Kennedy et al., 2016), Brainlife.io (Hayashi et al., 2024), and Zenodo (European Organization for Nuclear Research & OpenAIRE, 2013) offer a comprehensive ecosystem designed to promote open and reproducible neuroscience research. For instance, Brainlife.io is a cloud platform that supports data standardization, management, visualization, and processing, enabling automatic tracking of provenance history for thousands of data objects, thereby enhancing reproducibility and transparency (Krakauer et al., 2017). Platforms such as OpenNeuro specialize in data hosting services for MRI and EEG modalities, offering support for the BIDS format to ensure standardization and ease of use. NeuroLibre integrates Jupyter notebooks with manuscripts, allowing for interactive content. Platforms such as F1000Research support open publishing, enabling continuous peer review and immediate publication of datasets, software tools, and research findings. For a researcher, depending on the scale of a project, these platforms can help process, analyze, and visualize neuroimaging data at scale. For the larger community, the services offered by these platforms enable efficient dissemination of scientific artefacts. By embracing open platforms and tools, researchers can contribute to a paradigm shift in how data are analyzed, shared, and reused across various neuroscience disciplines, ultimately advancing the field and promoting collaborative efforts.

##### 8.4.1.1 Potential pain points and remedies

Each of the end-to-end workflow publishing platforms has a different learning curve and can be frustrating especially when you are at the end of an arduous publication process. Nonetheless, given the benefits to your future self and your peers, we recommend organizing small hackathons where several groups and laboratories can together invest some time and help troubleshoot the issues in this learning process.

##### 8.4.1.2 Takeaways

Think how the advent of cloud storage services has improved/rescued your digital life. Creating a complete cloud backup of your scientific hard work is very reassuring.

#### 8.4.2 Q2: data sharing (raw + derived)

Data, the heaviest of the research objects, typically require the most effort for sharing and are often published independently. Dedicated data-sharing portals such as OpenNeuro implement data standardization and validation protocols to facilitate data discovery, aggregation, and comparisons across studies, are recommended for large imaging datasets. At times, it is not possible to share the raw data due to privacy issues or technical limitations. However, it could still be possible to share the derived data or IDPs. In such cases, it would be more appropriate to jointly publish such data along with the accompanying code as described earlier.

When a study’s ethics approval does not include an “open-data” clause, usually, ECRs and PIs stop worrying about the data-sharing protocol. However, today, data sharing need not be fully open and centralized. Data sharing can be achieved with registered access or differential privacy and analysis can be federated. Such implementations often rely on semantic harmonization to enable data discovery. Thus, we should always prepare and publish harmonized metadata to enable data discovery and sharing based on local data governance policy and enable distributed, collaborative analysis.

Practical checklist for data publishing

1. De-identify your data by removing identifiable information (e.g. names, date of birth, face, and ears).
2. Use persistent identifiers such as DOIs to track your data releases and versions.
3. Publish a data dictionary covering all column names, missing value identifiers, and expected data ranges. Use controlled vocabulary when possible (seehttps://neuinfo.org/about/nifvocabulariesfor examples).
4. Choose an appropriate license for your data to enable their reuse within the relevant data governance framework. Use resources such ashttps://book.the-turing-way.org/reproducible-research/licensingandhttps://chooser-beta.creativecommons.org/to find a license that fits your use case.

##### 8.4.2.1 Potential pain points and remedies

Data comprising multiple data types, visits, and derived output can be challenging to share—especially if you have not invested time in earlier stages of standardization and curation. Nonetheless, as believers in and beneficiaries of open science, we all should strive to share data whenever possible. To proactively mitigate some of the data governance issues, one should pay close attention to the data consent and ethics forms and clearly define “personal identifiers”, “raw vs derived data” terms. Additionally, it can save a lot of time to “double-code” your participant identifiers early in the data collection as retrospectively renaming and de-identifying files after data processing becomes prohibitively difficult.

##### 8.4.2.2 Takeaways

It is only*fair*to use open data if you are willing to reciprocate!

#### 8.4.3 Q3: Sharing code and docs

Best practices for code writing, versioning, and sharing have been described extensively by many. The challenge in our experience is that neuroscientists usually have not had any structured software development training and hence their coding skills are shaped entirely on an as-needed basis using online resources. The field has recognized this gap over the years and now there exist several massive open online courses (MOOCs) for non-technical audiences (Chopra et al., 2023;Nichols et al., 2017).

One important lesson that we have learned ourselves through teaching such courses and organizing hackathons is that mental blocks for “coding” stem not primarily from mastering good python or R or Matlab scripts, but from the code documentation, versioning, testing, packaging, dependency management (i.e., environment), etc. These complementary, peripheral tasks tend to be more confusing and painful than searching syntax or debugging for coding errors. Thus, we recommend investing time in learning these interdependent steps to ensure reproducibility of your analysis and minimize future headaches for yourself!

Practical checklist to avoid common pitfalls

1. Start versioning your code at the very beginning, and not when the analysis is complete.
2. Package your code or share a file that details software dependencies (e.g. requirements.txt).
3. Document your code beyond README. See documentation (Step 3) section above.
4. Publish executable Jupyter notebooks with figures for quick overview of analysis.
5. Do not explicitly write usernames, tokens, and passphrases in your code. The AI bots will remember them!
6. Avoid using and storing absolute paths in your code. The AI bots will reveal them to others.
7. Use “pre-commit hooks” for your code to help with formatting, Codespell, avoid secrets leaks, and large data dumps to Git.
8. Choose an appropriate license for your code—seehttps://choosealicense.com/for an overview of open-source licenses and their specific restrictions.

##### 8.4.3.1 Potential pain points and remedies

For many, sharing your code can quickly trigger imposter syndrome to surface—that is difficult to deal with. However, a good therapeutic practice of documenting and unit testing in parallel as you write your code can greatly reduce the stress and uncertainty induced by the retrospective code refactors. To avoid anxiety associated with public sharing, we suggest starting with participating in hackathons, playing with private repos with your colleagues, and steadily building your collaborative coding skills.

##### 8.4.3.2 Takeaways

Sharing code is critical for reproducing your findings. It is hard to trust someone’s scientific claims based on a secret software recipe!

#### 8.4.4 Q4: manuscripts

The choice of publisher and the journal for your manuscripts depends on your research domain as well as preferences of your laboratory and your peers. Over the years there has been increasing demand for and shift towards “open-access” publications which is encouraging; however, the economics of this avenue is a separate topic that is out of scope. Nevertheless, we suggest the following best practices for the manuscript publication process.

1. Submit a pre-registration for your intended study before acquiring the results. Several journals offer this option for original research articles.
2. Consider Registered Reports (RRs) as an alternative to pre-registration. RRs include peer review before data collection, helping refine study design and reduce publication bias. They are supported by frameworks such as the Center for Open Science’s RR initiative (https://www.cos.io/initiatives/registered-reports) and the Peer Community In Registered Reports (PCI-RR) (https://rr.peercommunityin.org/), with some platforms offering journal-independent reviews.
3. Release an early copy on a preprint server (e.g. bioRxiv, medRxiv, OSF, Brainlife) to get feedback from wider community and increase visibility.
4. Prioritize journals with open-access options, low article processing charges (APCs), and that are from universities, scientific societies, or not for profit, when possible. Several journal options exist that waive or reduce APCs for researchers from low-or-middle-income countries (LMICs).
5. Link data DOIs, code repository releases, and documentation websites in the manuscript.

##### 8.4.4.1 Potential pain points and remedies

Science communicating is an underappreciated and time-consuming topic that is not the core competency of most scientists. It is, therefore, important to get community feedback soon to actively revise and refine your manuscript, and not completely rely on the fact checking skills of a busy set of reviewers with sample size of two or three. With certain caveats, it can be useful to consult AI tools for helping with the brainstorming and revising phases of the manuscript.

##### 8.4.4.2 Takeaways

Good writing is rewriting!

## 9 The Cost–Benefit Tradeoffs

At this point, one might be wondering how much additional work is needed to transition to the prescribed best practices in this guide. It is a fair question! Improving FAIRness of your research data and methods comes at a cost (Poldrack, 2019). As mentioned earlier, significant time investment, depending on individual skill sets, might be needed for adopting curation standards, learning new tools, and documenting your code and data. Then there is a potential fear of being scooped or losing competitive advantage from sharing research and knowledge. Having gone through these dilemmas ourselves during our five stages of grief, we note the following two practical approaches to tackling these questions. First, for ECRs planning a career in academia, many of these best practices turn out to be in your self-interest in the long term. Both from the perspective of gathering large sample sizes and improving efficiency of methodological iteration (e.g., applying new processing or analysis pipeline), the investment into the adoption of tools and practices from this guide can prove to be a huge time saver. Second, although we do support “open science,” we acknowledge the ethical protections and unequal access to resources in the global research community, which can dictate data-sharing policies. Nonetheless, the recommended practices hold true regardless of whether one shares data publicly, in a restricted consortium, or just with close collaborators and laboratory members. Today’s neuroimaging research activities are inescapably collaborative and as the scales and scopes grow larger in the foreseeable future, investing in and equipping ourselves with tools to handle future research setups are a wise investment.

## 10 Discussion

Like science itself, the open-science initiative has witnessed a significant evolution expanding the scope (Thibault et al., 2023) and availability of toolkits and platforms. This evolution underscores a shifting paradigm in how research is conducted, shared, and valued. Here, we attempted to provide end-to-end navigation guidelines to help newcomers, and ECRs make practical decisions related to open and reproducible research adoption. We highlighted common challenges associated with data curation, processing, harmonization, and publishing and described our approaches for mitigating these preemptively to avoid future headaches. Our solutions are meant to serve as a starting point, a template standard operating procedure that could be customized for individual needs with experience.

Admittedly, this self-therapeutic exercise mostly focuses on practical advice at an individual level. There remain several institutional challenges related to data-ethics, data-governance, and data-inclusivity that warrant everyone’s attention. The results from our survey (see Supplementary Materials:Figures S1, S2, andBox 2), albeit limited by sample size, do offer some insights and provide a baseline for a discussion on these systemic issues and desired resolutions by the community. Consolidating the survey feedback with our experiences, we advocate for three key broader changes to incentive structures relating to funding bodies, publishing journals, and academic institutions to build a more transparent and credible scientific ecosystem.

Box 2. **Survey highlights**

(A) Time burden of data wrangling: A lot of time is spent on organization and processing compared with annotation and publication.

(B) FAIRness of the data used (collected by others): Data are*findable*either through online search or through collaborator network. Fully*accessible*, open datasets are in the minority. Most datasets are semi-open. The*interoperability*of datasets was poor with only 35% cases using data dictionaries with some standardization. Data were reusable in ~ 50% of cases and too messy for reuse for a variety of reasons in other cases. Note that this does not take “dark data” silos (i.e., unpublished/undisclosed data sources) into account

(C) FAIRness of my collected data: High findability and accessibility of data but low findability, accessibility of metadata.

(D) Challenges preventing reproduction of published works: Poor findability of metadata, processing, and quality checks. Followed by difficulty in interoperability of data dictionaries.

(E) Paradox of reproducibility timeframes: Others can quickly reproduce my work (1 week—1 month) but I find it hard to reproduce the work by others (1–6 months).

## 11 Enhancing Data Discoverability and Accessibility

First, the data-sharing requirements, tools, and training need to be extended to include harmonized metadata to ensure findability, improve accessibility, and simplify interoperability. No more archived data dumps and subsequent investigative data forensics! Today, completely open data are still rare and there are two other kinds of neuroimaging datasets that ought to be accommodated to improve data discovery. First kind includes a long tail of several small, private, globally dispersed datasets, and the second consists of several medium-to-large-sized semi-open or registered access datasets (Madan, 2022). We encourage owners of both kinds to adopt community standards for data curation and openly publish harmonized metadata (see Step 4 for available tools), that is, inventory of the*available*data, to enable individual-level queries and counts without violating any privacy constraints. This would encourage cross-data-silo collaborations leading to more inclusive datasets with necessary data usage agreements.

## 12 Redefining Research Metrics

Second, the overemphasis on traditional publishing that is strongly incentivized by funding and career growth mechanisms bitterly summarized in a common aphorism: “*publish or perish,”*needs to be recalibrated with alternative means to evaluate research contributions, credit assignments, and training paradigms. As Goodhart’s law states:*“When a measure becomes a target, it ceases to be a good measure.”*This very much applies to the current overproduction of “original” research articles and their inflated valuations that often fail to deliver scientific and societal dividends. Adopting a more nuanced metric that values datasets and software as critical research outputs will help realign academic incentives with the goals of (open) science. As a remedy, we propose that FAIR dataset and neuroinformatic software describing publications ought to be valued higher as research contributions, similar to scientific articles (Puebla et al., 2024). Moreover, we hope that the academic hiring committees will restructure their assessment criteria with a more balanced rubric for rewarding investments of time and resources into adopting and facilitating openness and reproducibility.

## 13 Instituting Data Management Roles

Finally, we need to acknowledge that with great data (aka statistical power) comes great responsibility. The creation of a hierarchy of roles covering a variety of neuroinformatics responsibilities starting from data managers, data protection officers, and research software developers to chief technology officers within laboratories and institutions can help set up and maintain data governance, curation, and distribution. In the long term, these roles not only ensure the integrity and accessibility of research data but also support the researchers in adhering to best practices in data stewardship. In the era of big-data research, it is infeasible for a young trainee to carry out tasks requiring significant technical and scientific expertise. Often these efforts are unnecessarily duplicated with arevolving lab-door* compounding the messiness. Dedicated and sustainable roles would be a much more efficient and reliable solution for continuous data streams that need to be curated, processed, and published systematically.

We conclude this cathartic exercise by pointing readers to a letter published in Science—“Chaos in the Brickyard” (Forscher, 1963) written six decades ago by Bernard Forscher expressing concerns about deteriorating quality of scientific research and a flawed short-sighted approach by its suiters. Incredibly, this is prior to the advent of chatty LLMs, proliferation of journals, availability of HPCs, the Internet, and powerful open-source computing software and hardware. These advances have undoubtedly empowered researchers and made life-changing discoveries in neuroscience, but they are also contributing to the chaos at an accelerating pace. Therefore, here we have strategically focused on at-risk individuals susceptible to these maladies in early stages of academic trajectories. We hope, at least, this intervention reminds us to pause and recalibrate our scientific priorities in ways to motivate the research culture that prizes openness, reproducibility, and more crucially disincentivizes chaos.

## 14 Limitations and Future Directions

We note that the scope of this guide has several limitations. We do not cover the challenges related to data acquisition, which is an important part of the research cycle. The topics and tasks addressed in this work are focused on MRI and EEG and exclude other neuroimaging modalities. Our survey results are based on a relatively small sample size, and although they provide a very useful starting point estimate of the perceived challenges, the insights are possibly biased and can benefit from a larger sample. Future work should aim to expand surveying to a wider audience for better calibration of scientific challenges and priorities and assist with the adoption of open-science practices in other fields of neuroscience.

## Disclaimer

We, as early career researchers (ECRs) authors ourselves, conceptualized this article to be a recommendation piece by ECRs for ECRs. There exist several high-level articles, guides, and how-to tutorials on best practices targeted at PIs and new trainees. We noted a training gap for the transition phase between these academic roles which we decided to focus on explicitly. Nonetheless we believe the guide can be useful to researchers at different stages of their careers and encourage their engagement and feedback. We have written this guide based on our own frustrating encounters with the practical challenges and thus we have chosen a lighthearted tone and aphorisms to keep our own spirits up and to make it fun to read. We have marked these occurrences in-text by * and included an aphorism-data dictionary in theSupplementary Material, staying consistent with the best practices recommended here.

## Supplementary Material

Supplementary Material

## Data Availability

All datasets and code used in this study are made available to the research community. Detailed information regarding access to the data and the processing code is included. We have deposited the datasets in a publicly accessible repository (https://osf.io/2vznk/) and the code is hosted on GitHub (https://github.com/neurodatascience/ecr-fair). Links to these resources are provided for transparency and to facilitate reproducibility of our findings.
