# Supplementary material for: Challenging the status quo: A guide to open and reproducible neuroimaging for early career researchers
Source: Imaging Neurosci (Camb). 2025 Jun 24;3:IMAG.a.21. doi: 10.1162/IMAG.a.21 (PMC12319985; doi:10.1162/IMAG.a.21)
Supplement: Supplementary Material [file imag.a.21_supp.pdf]

Supplementary Material

1. The Gap Analysis Survey

To identify current challenges in daily research-related tasks, we surveyed the neuroimaging community (https://forms.gle/QMZa8KM53LJxKFJx7). We posted the survey on Neurostars and distributed it through our academic networks, mailing lists and social media to assess awareness of the issue, familiarity with the neuroinformatic tools, and specific pain points. Here are the summary findings of the survey (responses n=42). We note that the survey sample is relatively small and biased towards researchers interested in FAIR principles and neuroinformatic tools. Therefore, we expect the insights from the results would provide an optimistic estimation of the current challenges.

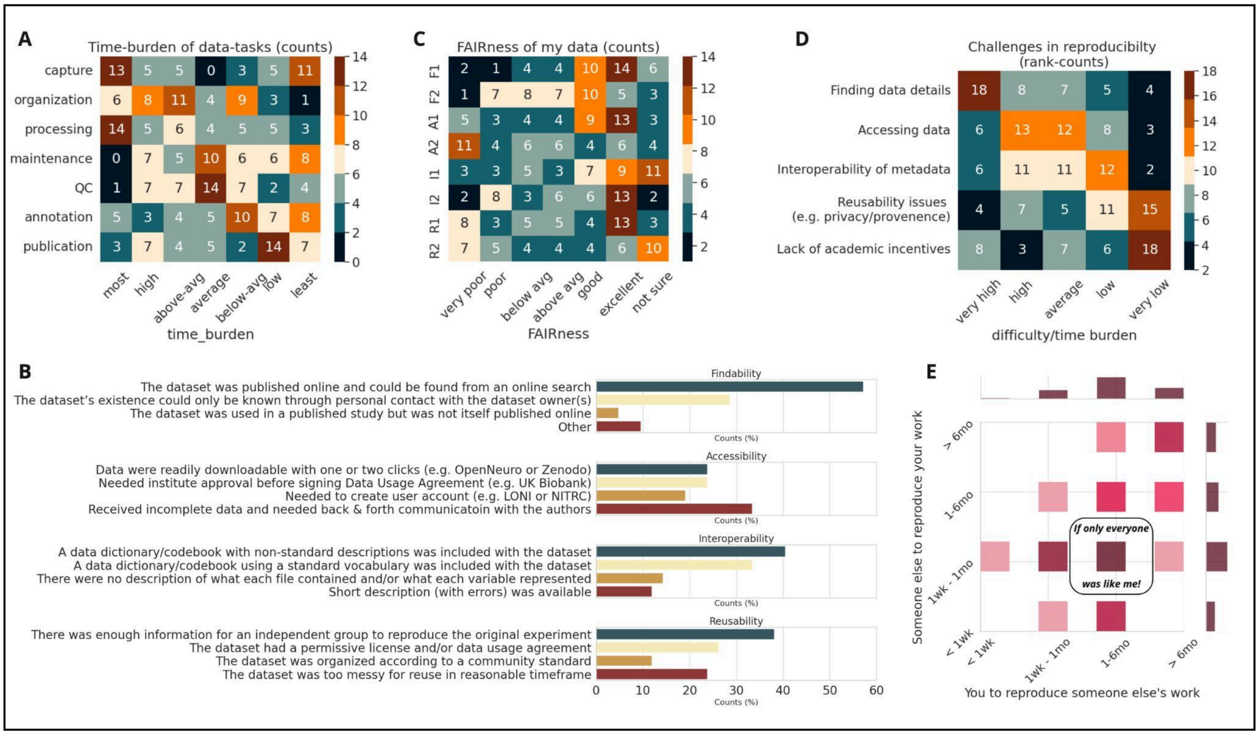

Figure S1: Survey insights

A) Time burden of research tasks: A lot of time is spent on data organization and processing compared to data annotation and publication.

B) FAIRness of the data used (collected by others): Data are *findable* either through online search or via collaborator network. Fully *accessible*, open datasets are in minority. Most datasets are semi-open. The *interoperability* of datasets were poor with only 35% cases using data dictionaries with some standardization. Data were reusable in ~ 50% of cases and too messy for reuse for a variety of reasons in other cases. Note that this does not take "dark data" silos (i.e. unpublished / undisclosed data sources) into account.

C) FAIRness of my data: High findability and accessibility of data. Low accessibility of metadata. Bimodal distribution for other dimensions with high uncertainty regarding metadata prevalence - often necessary for data reuse.
D) Challenges preventing reproduction of published works: Poor findability of data, processing, and quality checks. Followed by difficulty in accessing data and code as well as interoperability of data dictionaries.
E) (Dis)joint distribution of expected timeframes for "you reproducing someone else's work" vs. "someone else reproducing your work". The estimates for the former peaks at 1-6 months compared to 1week - 1month for the latter.

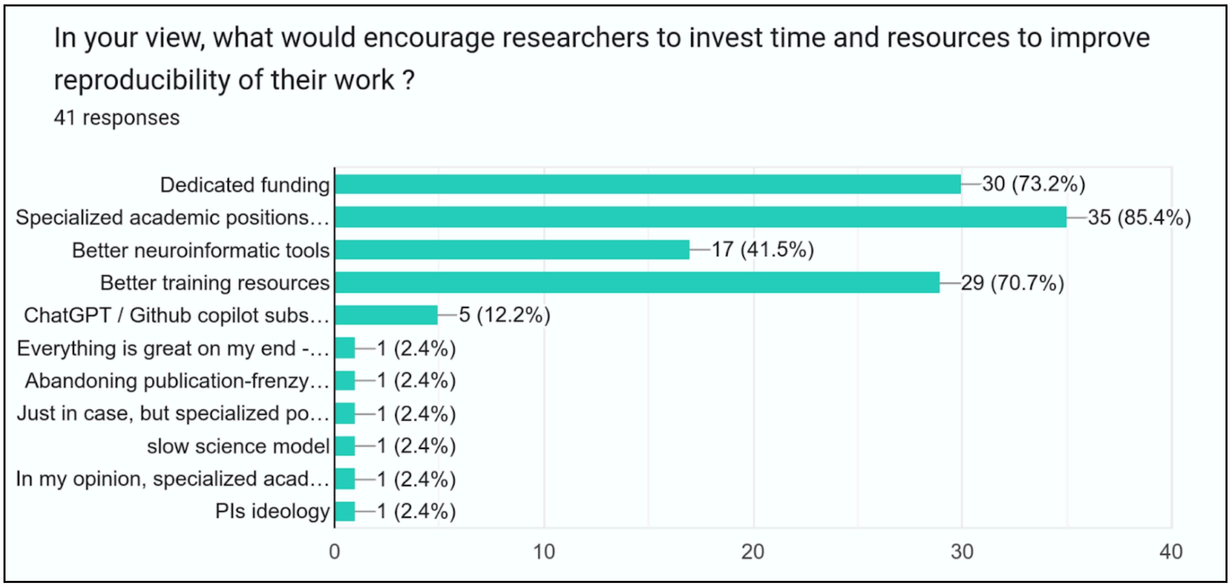

Figure S2: Survey results on the question of long-term solutions needed for improving reproducibility of their research.

Box S1: Survey highlights (N=42)

1. **Survey participants:**
   a. Career stage: ECR: 21, Grad students: 10, Principal Investigator: 9, other: 2
   b. Sex: 55% male, from 12 different countries
   c. Top 3 research domains: sMRI: 32, fMRI: 29, clinical/behavior: 18
2. **Awareness**
   a. People are aware of and highly concerned about the reproducibility crisis, with 95% rating it six or higher and 65% rating it eight or higher out of 10.
   b. FAIR principle awareness is poor, with only 52% of respondents reporting familiarity.
   c. Apart from BIDS no other neuroinformatic resource for data curation (e.g. DataLad, RedCap), processing (e.g. BIDS apps, FAIRly Big,, Boutiques, Containers), and publishing (e.g. OpenNeruo, Zenodo, OSF etc) is popular or being used regularly.
3. **Things that are working**
   a. Many public (semi)-open datasets are easy to find and access raw data.
   b. The reusability of public datasets is pretty good - 71% of participants reported the use of (semi) open datasets.
4. **Day-to-day challenges**
   a. Data curation and processing are most time-consuming. Data collection tasks have a U-shaped bimodal distribution likely from a split of research groups that collect data vs labs that use open/shared data.
   b. Data interoperability is poor due to unavailable data descriptions. For shared, semi-open data, more than half reported a lack of standard data dictionary or any data dictionary at all.
   c. People estimate a minimum requirement of several months to reproduce someone else’s work (data processing + analysis) that they have cited in their own papers.
   d. Details of data and method provenance, along with quality checks are hard to find or are missing.
   e. Documentation is typically done informally and written for one's own needs and, not written for sharing or publishing. Free form text files Readme / tabular data dictionary are the most preferred way of documentation and metadata. People are not familiar with tools that would standardize and openly share the documentation with others.
5. **Long-term challenges**
   a. Perceptional contradiction: Although reproducing published work of one’s peers was reported to be highly challenging, FAIRness of one’s own data and analysis was self-reported as very high (with the exception of accessibility of their metadata descriptors). This reveals the mismatch between the expectations and efforts invested by the same researchers in data consumers vs. data producers roles.
   b. Need for privacy-conscious tools: Open Data are impactful, but there is a growing long tail of valuable closed or restricted access data. This highlights the need for privacy aware data-sharing and metadata harmonization tools to facilitate collaborative analysis in federated setups.
   c. Institutional changes: Dedicated funding and specialized academic positions at the institutional level were the top suggestions for improving the reproducibility of research.

Based on the survey results, a pressing need for user-friendly, privacy-conscious tools is evident, particularly to ensure interoperability across 'open' datasets. Another notable gap emerges in the misalignment between open science practices and publishing platforms. The publishing process still revolves around text-based articles with little incentive, awareness and use of platforms for sharing (meta)data, code, and additional research outputs alongside manuscript submissions. While platforms such as Authorea, The Journal of Open Source Software, and BrainLife have taken laudable steps towards mitigating this gap, more user-friendly feature development and training are needed for embedding such tools in standard research practices.

2. MRI GUI software

Software	Description	Best For
FSL	Open-source software for analyzing and visualizing functional, structural, and diffusion MRI data.	Comprehensive MRI analysis including pre-processing, statistical analysis, and data visualization.
SPM	MATLAB-based software for processing fMRI, PET, and other neuroimaging data.	Advanced statistical modeling and brain mapping.
FreeSurfer	Open-source software suite for processing and analyzing human brain MRI images.	Structural MRI analysis including cortical reconstruction, volumetric segmentation, and morphometry.
MRIcroGL	Open-source visualization tool for viewing brain imaging data and applying overlays.	Quick visualization and conversion of neuroimaging data in various formats (NIfTI, DICOM, etc.).
BrainVoyager	Commercial software for fMRI, DTI, and EEG/MEG data analysis with a user-friendly GUI.	Multi-modal analysis of fMRI, DTI, and EEG/MEG with an intuitive interface.
AFNI	Open-source software for processing and analyzing fMRI data, with a variety of GUI and command-line tools.	Flexible fMRI processing and statistical analysis, with GUI and script-based options.
ANALYZE	Commercial software for visualizing and analyzing MRI and other medical imaging modalities.	Medical image analysis in clinical settings, with support for various imaging modalities.
3D Slicer	Open-source platform for 3D visualization and analysis of medical imaging data.	Versatile 3D imaging visualization, surgical planning, and advanced research tools across multiple modalities.
OsiriX	Commercial software for viewing and analyzing medical images, supporting DICOM and other medical formats.	Medical image visualization and analysis, primarily used in clinical and surgical planning settings.

Table S1: **MRI GUI-Based Software Comparison**. A detailed comparison of MRI GUI-based software tools, including both commercial and open-source options, and their suitability for various MRI analysis tasks.

3. Aphorism data dictionary

We have written this guide based on our own frustrating encounters with the practical challenges and thus we have chosen a lighthearted tone and aphorisms to keep our own spirits up and to make it fun to read. We have marked these occurrences in-text by *, which are further explained and contextualized here.

Phrase	Meaning	Research Context
“Rabbit holes”	This is a metaphor that describes the situation that involves getting lost while searching for answers for indefinite time.	We are using the metaphor to describe researchers browsing through Internet resources in an inefficient manner and often losing a lot of time before arriving at useful information.
“Data passengers”	We are referring to various data-samples (e.g., scans) as “passengers” embarking on a long journey (i.e. data wrangling tasks)	The metaphor of data passengers is related to our recommendation to use a “manifest” file in research workflows. This is inspired by real-life travel scenarios where “manifests” are used to track passengers on planes and ships throughout their journey.
“Long-haul data processing flights”	This analogy refers to compute heavy pipelines that often take high-performance clusters and several hours to complete processing.	This is a continuation of the “data passenger” metaphor, where these data samples go through long durations of processing on clusters or clouds. We are making a case for proactive checks and tracking of these tasks given their resource intensive nature.
“Leaky wallet”	A leaky wallet or leaky bucket implies loss of useful resources due to lack of proper tools or vigilance.	We consider data as a high-value asset. We borrow the “leaky wallet” analogy from the financial field to motivate better practices and vigilance to reduce preventable loss.
“Play 20 questions”	“Twenty questions” is a game where players ask yes or no questions to guess a word or object. The goal is to guess the word or object in 20 questions or fewer.	This is a painful exercise often a new student goes through usually via long thread emails to figure out answers to ambiguous data files and documentation.
“Statistical random walks”	A random walk is a stochastic process that describes a path comprising a succession of	The deliberate qualification of “statistical” random walk is to describe unfortunate trial-and-error based exploration of statistical

	random steps on a mathematical space. Here such space refers to the multiple statistical analytic choices explored by the researchers.	approaches, and methods hoping to find significant results.
“Revolving lab-door”	“Revolving-door” is used to refer to a situation in which the same events or problems recur in a continuous cycle.	Students graduate and leave the lab every year. With that often the research output is lost due to lack of training, knowledge transfer, and effort spent on standardization of data management practices.

Table S2: Aphorism data dictionary
